# Candidate Gene Analysis Reveals Strong Association of *CETP* Variants With High Density Lipoprotein Cholesterol and *PCSK9* Variants With Low Density Lipoprotein Cholesterol in Ghanaian Adults: An AWI-Gen Sub-Study

**DOI:** 10.3389/fgene.2020.456661

**Published:** 2020-10-30

**Authors:** Godfred Agongo, Lucas Amenga-Etego, Engelbert A. Nonterah, Cornelius Debpuur, Ananyo Choudhury, Amy R. Bentley, Abraham R. Oduro, Charles N. Rotimi, Nigel J. Crowther, Michèle Ramsay, AWI-Gen and H3Africa, H3Africa

**Affiliations:** ^1^Navrongo Health Research Centre, Navrongo, Ghana; ^2^Sydney Brenner Institute for Molecular Bioscience, Faculty of Health Sciences, University of the Witwatersrand, Johannesburg, South Africa; ^3^Division of Human Genetics, National Health Laboratory Service and School of Pathology, Faculty of Health Sciences, University of the Witwatersrand, Johannesburg, South Africa; ^4^West African Centre for Cell Biology of Infectious Pathogens, Department of Biochemistry, Cell and Molecular Biology, University of Ghana, Legon, Ghana; ^5^Julius Global Health, Julius Center for Health Sciences and Primary Care, University Medical Centre Utrecht, Utrecht University, Utrecht, Netherlands; ^6^Center for Research on Genomics and Global Health, National Human Genome Research Institute, National Institutes of Health, Bethesda, MD, United States; ^7^Department of Chemical Pathology, National Health Laboratory Service and School of Pathology, Faculty of Health Sciences, University of the Witwatersrand, Johannesburg, South Africa

**Keywords:** candidate gene, lipid, single nucleotide variant, Ghanaians, AWI-Gen

## Abstract

Variations in lipid levels are attributed partly to genetic factors. Genome-wide association studies (GWASs) mainly performed in European, African American and Asian cohorts have identified variants associated with LDL-C, HDL-C, total cholesterol (TC) and triglycerides (TG), but few studies have been performed in sub-Saharan Africans. This study evaluated the effect of single nucleotide variants (SNVs) in eight candidate loci (*ABCA1*, *LCAT*, *LPL*, *PON1*, *CETP*, *PCSK9*, *MVK*, and *MMAB*) on lipid levels among 1855 Ghanaian adults. All lipid levels were measured directly using an automated analyser. DNA was extracted and genotyped using the H3Africa SNV array. Linear regression models were used to test the association between SNVs and log-transformed lipid levels, adjusting for sex, age and waist circumference. In addition Bonferroni correction was performed to account for multiple testing. Several variants of *CETP*, *LCAT*, *PCSK9*, and *PON1* (MAF > 0.05) were associated with HDL-C, LDL-C and TC levels at *p* < 0.05. The lead variants for association with HDL-C were rs17231520 in *CETP* (β = 0.139, *p* < 0.0001) and rs1109166 in *LCAT* (β = −0.044, *p* = 0.028). Lower LDL-C levels were associated with an intronic variant in *PCSK9* (rs11806638 [β = −0.055, *p* = 0.027]) and increased TC was associated with a variant in *PON1* (rs854558 [β = 0.040, *p* = 0.020]). *In silico* functional analyses indicated that these variants likely influence gene function through their effect on gene transcription. We replicated a strong association between *CETP* variants and HDL-C and between *PCSK9* variant and LDL-C in West Africans, with two potentially functional variants and identified three novel variants in linkage disequilibrium in *PON1* which were associated with increasing TC levels in Ghanaians.

## Introduction

Cardiovascular disease (CVD) is a major health risk accounting for over 17 million deaths (about 30% of all deaths) globally each year. A major proportion (80%) of these deaths occurs in low and middle-income countries with the total number of annual deaths expected to reach 23.6 million by 2030 (Wang et al., 2016). One major risk factor for CVD is dyslipidemia which is a metabolic derangement that predisposes an individual to atherosclerosis (O’Donnell and Elosua, 2008; Reinikainen et al., 2015). There is variation in the prevalence of dyslipidemia across populations with the global adult prevalence of raised total cholesterol (TC) in 2008 estimated at 9.7% (WHO, 2011). The distribution of serum lipids is known to differ among individuals with African ancestry compared to better-studied world populations. These differences persist in populations of substantial African ancestry living in different environmental backgrounds, supporting the expectation that these differences in distribution are genetically determined (Willer et al., 2013; Bentley and Rotimi, 2017). It has been documented that approximately 25 to 80% of the inter-individual variation in lipid phenotypes is heritable (Perusse et al., 1997; Beekman et al., 2002). Single nucleotide variants (SNVs) in the following genes have been shown to be associated with differences in lipid levels: lipoprotein lipase (*LPL*) (Pirim et al., 2014), cholesteryl ester transport protein (*CETP*) (Ridker et al., 2009), paraoxonase 1 (*PON1*), (Durrington et al., 2001), ATP-binding cassette A1 (*ABCA1*) (Kelishadi et al., 2014), lecithin-cholesterol acyltransferase (*LCAT*) (Hovingh et al., 2005), protein convertase subtilisin/kexin type 9 (*PCSK9*) (Kotowski et al., 2006), methylmalonic aciduria cb1B (*MMAB*) (Sun et al., 2016) and mevalonate kinase (*MVK*) (Miao et al., 2017). Evidence from candidate gene and genome-wide association studies (GWASs) on the influence of genetic polymorphisms on lipid level variations comes primarily from studies in cohorts of European origin, and an increasing number in Asians and populations of African descent, but few in sub-Saharan Africans (Willer et al., 2013; Dron and Hegele, 2016). Identifying the common genetic variants associated with lipid levels in populations in Africa will assist in the detection of individuals at higher risk for dyslipidemia.

The ability to identify gene loci that associate with serum lipid (high density lipoprotein cholesterol [HDL-C], low density lipoprotein cholesterol [LDL-C], triglycerides [TG] and total cholesterol [TC]) levels has been advanced by the use of genotyping arrays that were developed from several projects including the HapMap project (The International HapMap Consortium et al., 2007; Wu et al., 2013), the 1000 Genomes Project (The 1000 Genomes Project Consortium, 2010; Wood et al., 2013) and the Genome of the Netherlands Project (Boomsma et al., 2014; van Leeuwen et al., 2015). These arrays are generally Eurocentric and under-represent common variants found in African populations. Our study is novel in that we used data from the H3Africa SNV array (Mulder et al., 2018) enriched with common SNVs in African populations and improved imputation using an African reference panel, to investigate the influence of SNVs at selected loci on lipid levels in our study population from Ghana.

The aim of our study was to examine the genetic association of SNVs and identify novel variants in the transcribed regions of eight genes previously associated with LDL-C, HDL-C, TC, and TG and to perform a replication study for the associated SNVs in another African cohort. This is the first African study to use an Afrocentric gene array to perform and replicate a candidate gene analysis of genetic associations with serum lipid levels.

## Materials and Methods

### Study Design and Population

This is a candidate gene study that was conducted in the Kassena-Nankana districts of northern Ghana as part of the Africa Wits-INDEPTH Partnership for Genomic research (AWI-Gen) project (Ramsay et al., 2016) under the broader Human Heredity and Health in Africa initiative (The H3Africa Consortium, 2014). The study population consisted of men and women aged 40–60 years who were selected using stratified random sampling from the two districts of northern Ghana. The east, west, north and south zones were selected and 25 clusters were randomly selected from each zone using data from the Navrongo Health and Demographic Surveillance System (NHDSS) (Oduro et al., 2012). A list of 2200 individuals (including 10% for non-response or refusal) was generated from the sampled clusters, with the sample size in each cluster being proportional to its population distribution in the age group 40 to 60 years. Individuals who agreed to participate in the study and who provided informed consent were assigned unique AWI-Gen identification numbers to ensure anonymity (Agongo et al., 2018).

Given this sample size, a power analysis was performed using Quanto software version 1.2.4 (Gauderman, 2002). Using information from a previous study that reported a mean HDL-C level in West Africans of 1.016 ± 0.321 mmol/l with an effect size of 0.388 mmol/l for the rs328 variant in the *LPL* gene at ∼5% minor allele frequency (MAF) (Bentley et al., 2014), we determined that our study had > 90% power to detect an effect size of at least 0.129 mmol/l for lipid traits at MAF > 0.05 for a sample size of 1800 individuals. This study was carried out in accordance with the recommendations of the National Institutes of Health guidelines, and approvals from the Human Research Ethics Committee (HREC) of the University of the Witwatersrand (ID No. M12109 and M170880), the Ghana Health Service Ethics Review Committee (ID No. GHS-ERC:05/05/2015) and the Navrongo Health Research Centre Institutional Review Board (ID No. NHRCIRB178) with community engagement and informed consent. All participants gave written informed consent in accordance with the approved documents.

### Demographic Data, Anthropometric Measurements and Lipid Analyses

Since many of the participants did not know their exact birthdates, their ages were mainly estimated using information from the Navrongo Health and Demographic Surveillance System (Oduro et al., 2012). Fasting whole blood samples were taken from participants for lipid measurements and DNA extraction for genetic analyses. Serum HDL-C, LDL-C, TC and TG were all measured directly using an automated chemistry analyser and waist circumference was measured as described in detail elsewhere (Ali et al., 2018).

### Selection of Candidate Genes

Genes were selected based on evidence from genome-wide association and candidate gene studies on lipid levels in peoples of African descent (Adeyemo et al., 2012; Elbers et al., 2012; Bentley et al., 2014) and sub-Saharan Africans (Abessolo et al., 2014; Niemsiri et al., 2015). We analysed polymorphic markers in 8 candidate genes: *ABCA1*, *LCAT*, *LPL*, *PON1*, *CETP*, *PCSK9*, *MVK*, and *MMAB* by selecting SNVs in the transcribed region of each gene (genome coordinates shown in Table 1).

**TABLE 1:** Number of variants tested within selected genes following quality control of data.

Gene symbol	Chromosome position	Start transcript	End transcript	Number of SNVs
*LPL*	8	19,759,228	19,824,769	286
*LCAT*	16	67,973,653	67,978,034	6
*CEPT*	16	111,682,249	111,727,724	117
*PON1*	7	94,926,988	94,954,019	94
*ABCA1*	9	107,543,283	107,690,518	632
*MVK*	12	110,011,060	110,035,922	55
*MMAB*	12	109,991,542	110,011,679	84
*PCSK9*	1	55,505,221	55,530,525	118

### DNA Extraction and Genotyping

The DNA was extracted using a modified protocol of the salting out method (Diego Chacon-Cortes, 2014). Briefly, this involved thawing the frozen samples before lysing the red blood cells with cold sucrose-Triton X lysing buffer. Following this was the centrifugation and washing of the pellets with the buffer. The white blood cells were lysed and proteins degraded by adding 20 mM TRIS 5 mM ethylenediaminetetraacetic acid (EDTA), 500 μl of proteinase-K and 200 μl of 10% sodium dodecyl sulphate. The lysate was precipitated by adding NaCl and centrifuging the solution. Absolute ethanol was added and agitated and the DNA spooled into a cryo vial. The DNA was washed in 70% ethanol to remove excess salt after which the DNA was suspended in 10 mM Tris, 1 mM EDTA buffer. Prior to genotyping the DNA samples were normalised by assessing the DNA concentration using a NanoDrop DN-100 spectrophotometer (Thermo Fisher Scientific, MA, United States). The ratio of the absorbance at 260/280 nm indicated the estimated DNA purity with an acceptable range between 1.8 and 2.0. A ratio below 1.5 was an indication of protein contamination. Genotyping was performed using the H3Africa single nucleotide variant (SNV) array designed on the Illumina platform. The array is enriched for common variation in African populations and contains >2.3 million SNVs (Mulder et al., 2018).

### Imputation and Quality Control Processes

The following pre-imputation quality control (QC) filters were applied to the genotyping data of the entire AWI-Gen dataset of 10,903 individuals from six different sites of which our study site is one. Individuals with a missing call rate >0.05 and SNVs with a missing call rate >0.05, MAF < 0.01 and Hardy-Weinberg equilibrium (HWE) *p*-value < 0.0001 were removed from the data set. SNVs that did not match the GRCh37 reference alleles or strands were also removed. The filtered dataset (with 1,729,661 SNVs and 10,903 individuals) was, pre-phased with EAGLE2 (Loh et al., 2016) and imputed using the Sanger Imputation Server with the African reference panel. The default Positional Burrows-Wheeler transform (PBWT) algorithm was used for imputation. At post imputation QC, poorly imputed SNVs (SNVs with IMPUTE2 information score < 0.6), SNVs with MAF < 0.01 and HWE *p*-value < 0.00001 were excluded resulting in the final quality controlled imputed dataset containing 10,903 individuals and 13,980,000 SNVs. The candidate gene data for this study was extracted from the larger dataset. The final data set included 1855 participants from Ghana. Only SNVs in the candidate genes with MAF > 0.05 in the regions described in Table 1 were used for data analysis in this study. To assess population structure, principal component analysis was done using the genotype data from the array.

### Data Analysis

Lipid levels were presented as medians with interquartile ranges and compared between men and women using Mann Whitney U test. The lipid levels were log-transformed and presented across genotypes of lead SNVs using analysis of covariance (ANCOVA) with adjustment for age, sex and waist circumference. PLINK version 1.90 was used for association analyses^1^ (Purcell et al., 2007; Chang et al., 2015). Data on SNVs in the transcribed regions of selected genes were extracted from the H3Africa AWI-Gen genotyped data for further analyses. Pearson χ^2^ test was used to assess deviation from HWE equilibrium by comparing observed to expected frequencies. Linear regression analyses were used to test for association between log-transformed lipid traits (Agongo et al., 2018) and SNVs. Standardised β values and confidence intervals were calculated using the major allele as reference. All *p*-values were corrected for multiple testing using the Bonferroni method (Bland and Altman, 1995) and adjusted for covariates (age, sex and waist circumference). LocusZoom plots were drawn using an online analysis tool at http://csg.sph.umich.edu/locuszoom by the University of Michigan, Department of Biostatistics, Centre for Statistical Genetics (Pruim et al., 2010). All Bonferroni-adjusted *p*-values at 5% significance level after covariate adjustment were considered significant.

### Functional Analysis of Significant SNVs

Functional variant analyses included localisation of the variant within the gene region and combined annotation dependent depletion (CADD) scores to predict the damaging effect of the variant on protein structure and function. Another annotation was loss of function tool (LoFtool) score which predicted genic intolerance and consequent susceptibility to disease. A CADD score above 10 implied a deleterious effect and a low LoFtool score indicated damaging effect of the mutations on the gene. The RegulomeDB (RDB) score (Boyle et al., 2012) was used to assess the regulatory potential of the variants. All of these were analysed using Variant Effect Predictor (McLaren et al., 2016). The 1000 Genomes database (accessed using^2^) was used to compare the MAFs of the SNVs analysed in the current study with those observed in other populations.

### Linkage Disequilibrium Among Significantly Associated Variants

Linkage disequilibrium (LD) among significantly associated SNVs was assessed using LDlink, a web-based tool (Machiela and Chanock, 2015).

### Replication

All variants with *p* < 0.05 in either the covariate-adjusted or unadjusted models (*n* = 21) were selected for replication using the Africa America Diabetes Mellitus study (AADM), which has been previously described (Rotimi et al., 2001). Briefly, AADM is a genetic epidemiology study of type 2 diabetes (T2D), enrolling participants from university medical centres in Nigeria (Enugu, Lagos, and Ibadan), Ghana (Accra and Kumasi), and Kenya (Eldoret). Analyses were conducted using linear mixed models of the log transformations of serum lipids in up to 4317 participants with available data. The first 2 PCs of the genotypes were included in the model. All models also included a genetic relationship matrix to account for the random effect of relatedness, as related individuals were included in AADM, and were adjusted for T2D, as this is a case-control study. Models were run using EPACTS (Efficient and Parallelizable Association Container Toolbox)^3^ with and without adjustment for additional covariates including age, sex, waist circumference and Bonferroni-corrected *p* values. Replication was defined as an association in a consistent direction with Bonferroni-corrected *p* < 0.0024 (i.e., 0.05/21).

## Results

### Characteristics of the Study Population

The demographic characteristics and lipid levels of the study population, stratified by sex, are shown in Table 2. Participants who had no genetic data were excluded from the analyses resulting in a sample size of 1855. The average age of the study participants was 51 years with women being significantly older than men (*p* = 0.0001). Waist circumference among women was significantly higher than that among men (*p* < 0.0001). There was no significant difference in LDL-C (*p* = 0.427), TC (*p* = 0.093) and TG (*p* = 0.854) levels between men and women but HDL-C levels were significantly lower (*p* = 0.0009) among women. Though not presented in the results, there was no self-reported dyslipidemia or self-reported lipid lowering treatment among the study participants. The genetic principal component analysis showed that there was no significant genetic structure that would influence the results, as illustrated in Supplementary Figure S1, and therefore genetic structure was not corrected for in the analyses.

**TABLE 2:** Basic characteristics of the study population in northern Ghana.

Variable	Men (*n* = 866)	Women (*n* = 989)	Total (*n* = 1855)	*p* value
Age (years)	50 (46–56)	52 (47–56)	51 (46–56)	0.0001
Waist circumference (cm)	72 (69–77)	75 (71–82)	74 (69–79)	< 0.0001
HDL-C (mmol/l)	1.14 (0.91–1.40)	1.08 (0.89–1.30)	1.11 (0.90–1.35)	0.0009
LDL-C (mmol/l)	1.60 (1.15–2.19)	1.61 (1.20–2.15)	1.61 (1.18–2.16)	0.6599
TC (mmol/l)	3.11 (2.59–3.70)	3.17 (2.68–3.78)	3.15 (2.62–3.75)	0.0929
TG (mmol/l)	0.56 (0.43–0.73)	0.55 (0.43–0.74)	0.56 (0.43–0.73)	0.8541

*Data given as median (interquartile range).*

### Variants Associated With Lipid Levels

We tested the association between variants within the transcribed regions of selected genes (*ABCA1*, *CETP*, *LCAT*, *LPL*, *MMAB*, *MVK*, *PCSK9*, and *PON1*) and each lipid fraction as a continuous variable (HDL-C, LDL-C, TC, and TG). There were associations between variants of *CETP*, *LCAT*, *PCSK9*, and *PON1* and lipid levels at *p* < 0.05 after adjustment for age, sex and waist circumference (Table 3). The lead associated SNVs were the following: rs17231520 and rs34065661 in *CETP* and rs1109166 in *LCAT* for HDL-C, rs11806638 in *PCSK9* for LDL-C and rs854558 in *PON1* for TC. For *CETP*, there were 11 SNVs that were significantly associated with HDL-C levels, with the strongest associations being observed at rs17231520 and rs34065661 (Table 3). All but one of these SNVs (rs3816117) showed a positive association between the minor allele (relative to the major allele) and HDL-C levels. One SNV within the *LCAT* gene (rs1109166) showed a significant negative association with HDL-C levels. With regards to LDL-C, 4 SNVs within the *PCSK9* gene were identified with significant associations with serum LDL-C levels. However, only one of these (rs11806638) remained significant (*p* < 0.05) after adjustment for age, sex and waist circumference. All these variants demonstrated negative associations with LDL-C levels. Five SNVs in the *PON1* gene were significantly associated with serum TC levels, but only 3 (rs854558, rs854564, and rs854565) of these remained significant after adjustment for age, sex and waist circumference. All these SNVs were positively associated with TC serum levels. No significant gene associations were found for serum TG levels.

**TABLE 3:** Association of single nucleotide variants of selected candidate genes with lipid levels among the total study group (*n* = 1855).

						Without covariates			With covariates^2^	
SNV(Gene)	A1/A2	Position^1^	MAF	pHWE	β (SE)	p_*R*_ value	p_*B*_ value	β (SE)	p_*R*_ value	p_*B*_ value
**HDL-C**										
rs17231520(*CETP*)	A/G	56995827	0.089	1.000	0.1386 (0.0209)	4.19e-11	4.90e-09	0.1387 (0.0209)	3.80e-11	4.44e-09
rs34065661(*CETP*)	G/C	56995935	0.090	1.000	0.1372 (0.0207)	4.47e-11	5.23e-09	0.1371 (0.0207)	4.30e-11	5.03e-09
rs711752(*CETP*)	A/G	56996211	0.246	0.573	0.0833 (0.0137)	1.35e-09	1.58e-07	0.0845 (0.0137)	7.45e-10	8.72e-08
rs708272(*CETP*)	A/G	56996288	0.245	0.531	0.0832 (0.0137)	1.42e-09	1.66e-07	0.0844 (0.0137)	7.82e-10	9.14e-08
rs891142(*CETP*)	T/C	57003977	0.098	1.000	0.0984 (0.0200)	9.29e-07	1.09e-04	0.0983 (0.0200)	9.55e-07	1.11e-04
rs891143(*CETP*)	T/C	57003980	0.097	0.692	0.0976 (0.0200)	1.16e-06	1.36e-04	0.0973 (0.0200)	1.24e-06	1.45e-04
rs4784740(*CETP*)	G/C	57001085	0.091	0.780	0.1008 (0.0208)	1.35e-06	1.58e-04	0.1009 (0.0208)	1.31e-06	1.53e-04
rs3816117(*CETP*)	T/C	56996158	0.378	0.459	−0.0539(0.0124)	1.48e-05	1.73e-03	−0.0551(0.0124)	9.17e-06	1.07e-03
rs158478(*CETP*)	C/A	57007734	0.300	0.294	0.0487 (0.0129)	1.59e-04	0.01858	0.0488 (0.0129)	1.49e-04	0.01740
rs891141(*CETP*)	G/T	57003723	0.168	0.115	0.0576 (0.0157)	2.46e-04	0.02875	0.0589 (0.0157)	1.76e-04	0.02053
rs289719(*CETP*)	T/C	57009941	0.440	0.963	0.0430 (0.0120)	3.62e-04	0.04236	0.0443 (0.0120)	2.30e-04	0.02685
rs1109166(*LCAT*)	T/C	67977382	0.219	0.946	−0.0406(0.0145)	0.00510	0.03060	−0.0408(0.0145)	0.00476	0.02853
**LDL-C**										
rs11806638(*PCSK9*)	A/C	55518160	0.396	0.961	−0.0601(0.0152)	7.87e-05	9.29e-03	−0.0549(0.0149)	2.27e-04	0.02683
rs11804420(*PCSK9*)	G/A	55520445	0.298	0.346	−0.0612(0.0165)	2.02e-04	0.02389	−0.0513(0.0161)	0.00147	0.1736
rs11800231(*PCSK9*)	A/G	55517940	0.215	0.149	−0.0682(0.0184)	2.21e-04	0.02601	−0.0635(0.0180)	4.25e-04	0.05017
rs45545732(*PCSK9*)	T/A	55519231	0.273	0.860	−0.0605(0.0168)	3.08e-04	0.03634	−0.0521(0.0164)	0.00147	0.17400
**TC**										
rs854558(*PON1*)	T/C	94945374	0.385	0.659	0.0435 (0.0110)	7.40e-05	6.95e-03	0.0404 (0.0109)	2.13e-04	0.02005
rs854564(*PON1*)	G/T	94948182	0.386	0.807	0.0412 (0.0110)	1.76e-04	0.01654	0.0383 (0.0109)	4.68e-04	0.04398
rs854565(*PON1*)	A/G	94948344	0.386	0.807	0.0412 (0.0110)	1.76e-04	0.01654	0.0383 (0.0109)	4.68e-04	0.04398
rs854566(*PON1*)	A/G	94948749	0.377	0.401	0.0405 (0.0110)	2.22e-04	0.02090	0.0374 (0.0109)	6.23e-04	0.05858
rs854567(*PON1*)	A/G	94948784	0.377	0.401	0.0405 (0.0110)	2.22e-04	0.02090	0.0374 (0.0109)	6.23e-04	0.05858

*A1/A2, Minor/major allele; MAF, Minor allele frequency in the study population; pHWE, Hardy-Weinberg p value; N, total number of participants; SE, standard error; ^1^Genomic position and chromosome in build 37; p_R_, raw p value; p_B_, Bonferroni-adjusted p value; ^2^ Covariates were sex, age and waist circumference.*

**FIGURE 1:**
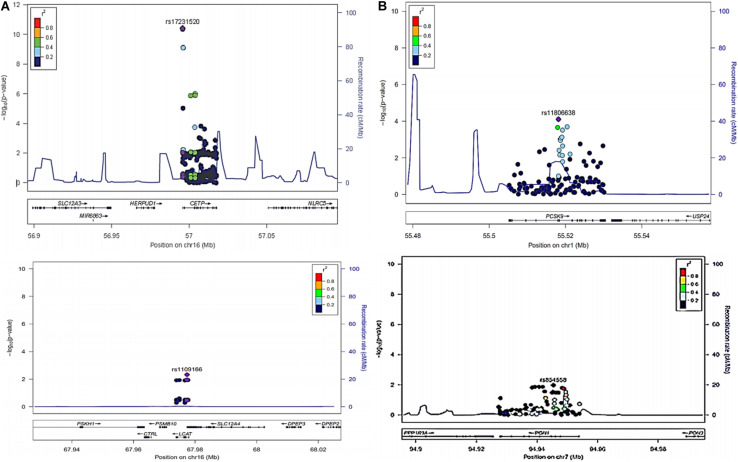
**(A)** LocusZoom plots of rs17231520 and rs34065661 (*CETP*) and rs1109166(*LCAT*) associated with higher and lower levels of HDL-C respectively after adjustment for sex, age and waist circumference. **(B)** LocusZoom plots of rs11806638(*PCSK9*) and rs854558(*PON1*) associated with lower levels of LDL-C and higher levels of TC respectively after adjustment for sex, age and waist circumference.

**FIGURE 2:**
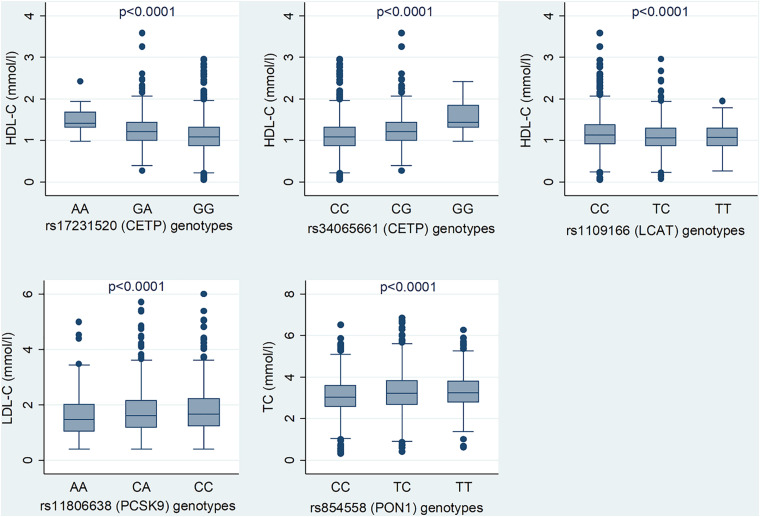
Median lipid levels for the genotypes of the lead SNVs among Ghanaian adults. The *p* value is adjusted for age, sex and waist circumference.

The LocusZoom plots for the significant associations are shown in Figures 1A,B and lead SNVs from *CETP*, *LCAT*, *PCSK9*, and *PON1* are shown. Figure 2 shows the median serum lipid levels given each genotype for each lead SNV. The rs17231520 SNV within *CETP* shows an additive effect, with serum HDL-C levels increasing in the presence of the minor allele (A). The SNV within *LCAT*, rs1109166, showed a dominant and negative effect of the minor allele (T) on serum HDL-C levels. The minor allele (A) at rs11806638 in the *PCSK9* gene demonstrated a negative effect on LDL-C levels, in a recessive manner. The serum levels of TC were positively associated with the minor allele (T) at rs854558 in the *PON1* gene, with the T allele acting in a dominant fashion.

### Functional Annotation of Significantly Associated Variants

Results of functional analyses of the significantly associated variants are summarised in Table 4. The functional annotation shows that the leading associated variant (rs17231520: *p* = 4.44e-09) in the *CETP* region was an upstream variant with an RDB score of 5 suggesting possible regulation of the rate of transcription. The second lead SNV in *CETP* (rs34065661: *p* = 5.03e-09) was a missense variant which also had an RDB score of 5 but with a CADD score > 10 which suggested a possible deleterious effect on gene structure and protein function. All other significantly associated variants were located in the introns of four genes (*CETP, LCAT, PCSK9*, and *PON1)* and had RDB scores of 4 or 5 which indicated possible effects on binding sites and regulation of transcription. The LoFtool score of all mutations in *PON1* was 0.787 suggesting a mild effect of these mutations to high TC levels. The LoFtool scores of rs1109166 in *LCAT* and rs11806638 in *PCSK9* were 0.127 sand 0.467, respectively, suggesting possibly damaging effects of the mutations on gene function.

**TABLE 4:** Functional annotations of lipid trait associated SNVs in Ghanaian adults and their minor allele frequencies in comparison to other population groups.

Variant(Gene)	Localisation	CADD score	RDB score	LoFtool score		Minor allele frequencies per population				
					Minor allele	Study population	Africa	Europe	East Asia	South Asia
rs17231520(*CETP*)	Upstream variant	4.38	5	0.970	A	0.089	0.082	0.000	0.000	0.000
rs34065661(*CETP*)	Missense variant	12.64	5	0.970	G	0.090	0.083	0.000	0.000	0.000
rs711752(*CETP*)	Intron variant	8.46	5	0.970	A	0.246	0.246	0.426	0.375	0.451
rs708272(*CETP*)	Intron variant	0.46	5	0.970	A	0.245	0.247	0.425	0.375	0.448
rs891142(*CETP*)	Intron variant	2.69	–	0.970	T	0.098	0.108	0.000	0.021	0.010
rs891143(*CETP*)	Intron variant	1.39	–	0.970	T	0.097	0.103	0.000	0.000	0.000
rs4784740(*CETP*)	Intron variant	0.86	6	0.970	G	0.091	0.096	0.000	0.000	0.000
rs3816117(*CETP*)	Intron variant	0.77	5	0.970	T	0.378	0.404	0.516	0.518	0.397
rs158478(*CETP*)	Intron variant	1.57	–	0.970	C	0.300	0.284	0.524	0.555	0.428
rs891141(*CETP*)	Intron variant	0.17	5	0.970	G	0.168	0.153	0.001	0.021	0.013
rs289719(*CETP*)	Intron variant	2.45	5	0.970	T	0.440	0.441	0.330	0.310	0.452
rs1109166(*LCAT*)	Intron variant	3.06	4	0.217	T	0.219	0.260	0.815	0.904	0.761
rs11806638(*PCSK9*)	Intron variant	3.86	5	0.467	A	0.396	0.356	0.064	0.027	0.064
rs854558(*PON1*)	Intron variant	1.92	–	0.787	T	0.385	0.340	0.714	0.702	0.614
rs854564(*PON1*)	Intron variant	3.01	5	0.787	G	0.386	0.341	0.286	0.299	0.387
rs854565(*PON1*)	Intron variant	2.23	–	0.787	A	0.386	0.341	0.286	0.298	0.387

*CADD, combined annotation dependent depletion score predicts damaging effect of the variant on protein structure and function; LoFtool, loss of function tool score predicts gene intolerance and consequent susceptibility to disease; RDB, RegulomeDB score assesses the regulatory potential of the variant; the frequencies are for the 1000 Genomes data.*

**FIGURE 3:**
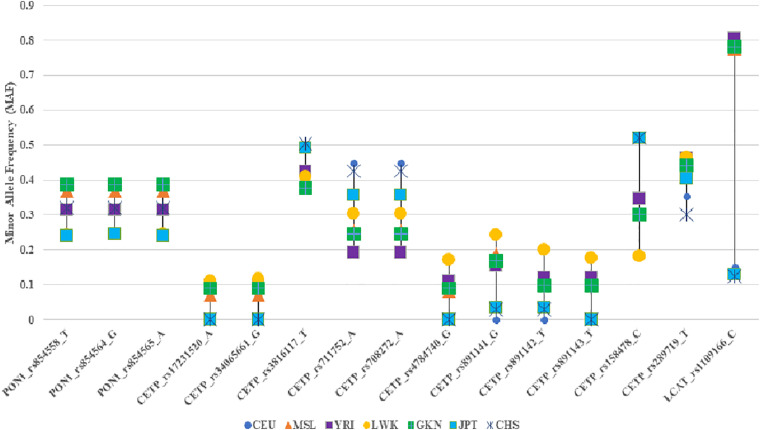
Comparison of the minor allele frequencies of the significantly associated SNPs in the study population with those of the 1000 Genomes. CEU: Northern Europeans from Utah, MSL: Mende in Sierra Leone, YRI: Yoruba in Ibadan, Nigeria, LWK: Luhya in Webuye, Kenya, GKN: the study population, JPT: Japanese in Tokyo, Japan, CHS: Southern Han Chinese.

The frequencies of the minor alleles for our lead SNVs were similar to what have been observed in 1KG African populations (Table 4 and Figure 3). Notably, some of our associated variants, including rs17231520 and rs34065661 in *CETP*, are observed only in those with African ancestry. For the SNVs that were common to Africans and European ancestry all the SNVs in the study population, except rs316117-T, rs711752-A, rs708272-A, and rs158478-C, had higher variant allele frequencies than those of the SNVs in the European ancestry populations.

### Linkage Disequilibrium Among Significant Variants

The assessment of LD among significantly associated SNVs was performed using LDlink, a web-based tool (Machiela and Chanock, 2015). The value of r^2^ in *CETP* ranged from 0.000 to 1.000 (Supplementary Table S1) while that of *PON1* ranged from 0.993 to 1.000 (Supplementary Table S2). In *CETP*, there was complete LD between two pairs of SNVs (rs711752 with rs708272 and rs17231520 with rs34065661) and strong LD between three pairs of SNVs (rs891142 with rs891143, rs4784740 with rs891142 and rs4784740 with rs891143) (Supplementary Table S1). All the significant variants in *PON1* were in complete LD with each other (Supplementary Table S2).

### Replication Results

Replication results of significant variants in our population using the AADM study are shown in Table 5. All the *CETP* variants before and after adjustment for covariates, except rs158478, were replicated with the two lead signals in the replication analyses, after adjustment for covariates, being rs34065661 (β = 0.109, *p* = 5.20e-10) and rs17231520 (β = 0.107, *p* = 1.52e-10). Similarly, all signals in *PCSK9* before adjustment for covariates were replicated, and the rs11806638 variant after adjustment for covariates was replicated (β = −0.031, *p* = 0.00040). All replicated associations showed the same direction of allelic effect. None of the significant SNVs in *LCAT* and *PON1* was replicated.

**TABLE 5:** Replication results of lipid trait associated variants in East and West Africans (AADM study).

					Without covariates		With covariates^2^	
SNV(Gene)	A1/A2	Position^1^	N	MAF	β (SE)	*p* value	β (SE)	*p* value
**HDL-C**								
**rs17231520 (*CETP*)**	**A/G**	**56995827**	**4317**	**0.076**	**0.1063 (0.01726)**	**8.07e-10**	**0.1069 (0.01716)**	**5.20e-10**
**rs34065661 (*CETP*)**	**G/C**	**56995935**	**4317**	**0.077**	**0.109 (0.0171)**	**2.00e-10**	**0.1091 (0.017)**	**1.52e-10**
**rs711752 (*CETP*)**	**A/G**	**56996211**	**4317**	**0.224**	**0.06399 (0.01083)**	**3.76e-09**	**0.0638 (0.01077)**	**3.41e-09**
**rs708272 (*CETP*)**	**A/G**	**56996288**	**4317**	**0.223**	**0.0643 (0.01087)**	**3.54e-09**	**0.0641 (0.0108)**	**3.21e-09**
**rs891142 (*CETP*)**	**T/C**	**57003977**	**4317**	**0.096**	**0.0679 (0.0157)**	**1.6e-05**	**0.06801 (0.01562)**	**1.37e-05**
**rs891143 (*CETP*)**	**T/C**	**57003980**	**4317**	**0.088**	**0.07841 (0.01635)**	**1.68e-06**	**0.07826 (0.01627)**	**1.57e-06**
**rs4784740 (*CETP*)**	**G/C**	**57001085**	**4317**	**0.081**	**0.08446 (0.01681)**	**5.30e-07**	**0.08399 (0.01673)**	**5.40e-07**
**rs3816117 (*CETP*)**	**T/C**	**56996158**	**4317**	**0.426**	**−0.04769 (0.009266)**	**2.8e-07**	−**0.04784 (0.009212)**	**2.159e-07**
rs158478 (*CETP*)	C/A	57007734	4317	0.301	0.01886 (0.01012)	0.06234	0.01819 (0.01006)	0.07064
**rs891141(*CETP*)**	**G/T**	**57003723**	**4317**	**0.142**	**0.06963 (0.01308)**	**1.08e-07**	**0.06754 (0.01302)**	**2.24e-07**
**rs289719 (*CETP*)**	**T/C**	**57009941**	**4317**	**0.434**	**0.03065 (0.009093)**	**0.00076**	**0.02986 (0.009044)**	**0.00097**
rs1109166 (*LCAT*)	T/C	67977382	4317	0.236	−0.01591 (0.01109)	0.1516	−0.01629 (0.01103)	0.1399
**LDL-C**								
**rs11806638 (*PCSK9*)**	**A/C**	**55518160**	**4287**	**0.378**	−**0.03089 (0.008969)**	**0.00058**	−**0.03106 (0.008801)**	**0.00042**
**rs11804420 (*PCSK9*)**	**G/A**	**55520445**	**4287**	**0.253**	−**0.04537 (0.00981)**	**3.9e-06**	−0.04365 (0.009631)	6.0e-06
**rs11800231(*PCSK9*)**	**A/G**	**55517940**	**4287**	**0.197**	−**0.04148 (0.01084)**	**0.00013**	−0.04165 (0.01065)	9.3e-05
**rs45545732(*PCSK9*)**	**T/A**	**55519231**	**4287**	**0.228**	−**0.03805 (0.01023)**	**0.0002**	−0.03612 (0.01004)	0.00033
**TC**								
rs854558(*PON1*)	T/C	94945374	4317	0.355	0.009671 (0.006512)	0.1376	0.01005 (0.006332)	0.1125
rs854564(*PON1*)	G/T	94948182	4317	0.357	0.009167 (0.006488)	0.1578	0.009591 (0.006309)	0.1285
rs854565(*PON1*)	A/G	94948344	4317	0.357	0.008752 (0.006486)	0.1773	0.009207 (0.006308)	0.1445
rs854566(*PON1*)	A/G	94948749	4317	0.342	0.008547 (0.006536)	0.1911	0.009499 (0.006355)	0.1351
rs854567(*PON1*)	A/G	94948784	4317	0.342	0.009227 (0.006547)	0.1588	0.01017 (0.006366)	0.1103

*A1/A2, Minor/major allele; MAF, Minor allele frequency in AADM study; pHWE, Hardy-Weinberg p value; N, total number of participants; SE, Standard error; ^1^Genomic position and chromosome in build 37; ^2^ Covariates were age, sex, waist circumference and T2D; results of replicated variants indicated in bold.*

## Discussion

This study on adults in northern Ghana evaluated the associations between lipid levels and common nucleotide variants in the transcribed regions of eight candidate genes involved in lipid pathways. After adjustment for sex, age and waist circumference, SNVs in four genes (*CETP*, *LCAT*, *PCSK9*, and *PON1*) were significantly associated with lipid levels. Variants in *CETP* and *LCAT* were significantly associated with HDL-C levels. One variant in *PCSK9* was significantly associated with LDL-C and another in *PON1* associated with TC levels.

Previous studies have suggested that African populations and populations of African descent tend to have higher HDL-C levels than Europeans suggesting a more favourable profile for CVD risk (Seedat et al., 1992; Chaturvedi et al., 1994; Johnson et al., 2004; Miljkovic-Gacic et al., 2006; D’Adamo et al., 2010). In this study higher HDL-C levels were strongly associated with an upstream variant, a missense variant and intronic variants in the *CETP* gene. The lead SNV (rs17231520) was in the upstream regulatory region and functional prediction suggests an impact on the rate of transcription. The high CADD score of the associated missense variant (rs34065661) is interesting as it suggests a deleterious effect on protein function. The two lead SNVs in *CETP* may be the causal variants for high HDL-C in West Africans since they are functionally relevant, their effect sizes are larger than those of the others and they are common in African populations (>0.08), and were not observed in 1000 Genomes Project European and Asian populations. Additionally we identified several intronic SNVs in the *CETP* gene where the variant alleles were positively associated, except rs3816117, which was negatively associated, with HDL-C levels. Functional analysis suggests these intronic variants regulate the rate of gene transcription but their low CADD scores indicate that the effect of their regulatory activity on gene function is mild. The significantly associated variants in *CETP* probably impaired the ability of the gene to accelerate the transfer of cholesteryl esters (CE) from cholesteryl ester-rich HDL-C formed by *LCAT* to other lipoprotein particles resulting in increased HDL-C levels (Brown et al., 1989).

Most of the SNVs in the *CETP* gene replicate previous GWAS findings in peoples of African descent (Buyske et al., 2012; Elbers et al., 2012; Pirim et al., 2016). Our replication analysis, using the African American diabetes mellitus (AADM) study involving over 4000 East and West Africans (Rotimi et al., 2001), revealed that the direction and strength of effects of the SNVs in *CETP* were consistent, except for rs158478. The minor allele frequencies of the replicated variants in our population were similar to the allele frequencies in other Africans both in the 1000 Genomes data and replication population but the effect sizes of the variants were larger in our study (Tables 3, 5). The rs17231520 (A) and rs34065661 (G) alleles which are absent in European and Asian populations, were previously found to be positively associated with HDL-C levels among African Americans (Buyske et al., 2012; Elbers et al., 2012) and sub-Saharan Africans (Pirim et al., 2016). As in our study, the two variants were in strong LD in all these studies and the frequencies of the rs17231520 (A) and rs34065661 (G) alleles were similar to those in our populations but the effect sizes of these alleles were smaller in our study. Similarly our study replicated rs711752, rs708272, rs891141, rs891143 and rs289719 variants in *CETP* that were previously found to be associated with HDL-C in sub-Saharan Africans by Pirim et al. (2016). Though the MAFs of these variants in this African population were similar to those in our study, the effect sizes of the variants were generally larger in our population. Also, the MAFs of these variants in our population were similar to those in other African populations (MSL, YRI, and LWK) but were larger than the MAFs in Europeans and Asian populations (CEU, JPT, CHS).

In our study the minor allele (T) of rs1109166 in *LCAT* was negatively associated with HDL-C levels. Since the T allele is the common non-African allele (0.76 to 0.90 in Europeans and Asians), its association with low HDL-C in Africans may not be functionally relevant, despite the fact that the low LoFtool score predicts a deleterious effect for this intronic variant (Le Hir et al., 2003; Chorev and Carmel, 2012). It may be tagging other functionally relevant variants through LD, which could interfere with the ability of *LCAT* to form CE and to promote cholesterol efflux from peripheral cells. Furthermore, this variant was not associated with HDL-C levels in the replication study. Our study is therefore the first to report this association in an African population and requires further investigation.

PCSK9 binds to the LDL receptor (LDLR) to form a complex which moves from the endosomal recycling pathway to the lysosome for degradation (Akram et al., 2010). Since LDLR removes cholesterol-rich LDL particles from plasma (Poirier et al., 2008), a loss of function or reduction in PCSK9 activity inhibits LDLR degradation and leads to the reduction of LDL-C in the blood (Park et al., 2004). In this study the rs11806638 (A) allele in *PCSK9* was negatively associated with LDL-C level. In addition to this variant being previously associated with lower LDL-C levels in African Americans (Musunuru et al., 2012) we replicated its association in other sub-Saharan Africans from the AADM study (Rotimi et al., 2001). The replication of this variant has clinical translational impact since it has the potential to act as a *PCSK9* inhibitor in Africans with high LDL-C levels leading to reduced CVD risk.

*PON1* encodes an enzyme that is present in the HDL particle and protects HDL and LDL from peroxidation by degrading or hydrolysing specific oxidised cholesteryl esters and phospholipids contained in oxidised lipoproteins (Barter et al., 2004). This process contributes mainly to the antioxidant properties of the HDL particle which results in improved macrophage-mediated cholesterol efflux (Parthasarathy et al., 1990). Genetic variation in the gene is therefore associated with increased TC levels. Our results show positive associations of rs854558-T, rs854564-G, and rs854565-A alleles in *PON1* with TC in the study population. To the best of our knowledge this study is the first to report the association of these variants with TC levels, although other studies have shown associations of other SNVs in the *PON1* gene with serum lipid levels (Chang et al., 2010; Bentley et al., 2014; Luo et al., 2018). The similar association observed in the lead variant and other signals (rs854564 and rs854565) in the gene with TC levels could be due to the complete LD between these signals and the lead variant. Though the frequency of the variant allele of rs854558 is substantial (0.385) in the study population, its effect size is small and it is unlikely to play a major role in hypercholesterolemia and CVD risk.

### Strengths and Limitations of the Study

The study had several strengths. Firstly, individuals were genotyped using an array enriched for gene variants that are common in African populations and genotypes were imputed using and African reference panel. Secondly, it is one of very few studies to evaluate the association of SNVs with lipid levels in Africa thereby contributing knowledge on the influence of genetic factors on lipid levels in under-studied African populations. The study is the first of its kind to identify the association of three polymorphisms in LD (rs854558, rs854564, and rs854565) in *PON1* which were associated with higher TC levels. This population is relatively drug-naïve as they do not have easy access to lipid-lowering medication. The West and East African replication cohort (Rotimi et al., 2001) supported the association of *CETP* variants with high HDL-C levels and *PCSK9* variants with LDL-C levels.

In terms of limitations, the sample size was relatively small and therefore underpowered to detect variants with small effect sizes. However, power calculations suggested that there was sufficient power to replicate previously reported gene associations with lipids. Candidate genes were selected from studies involving people of African descent, but not resident in Africa, and thus genes chosen may not be appropriate for indigenous African populations. Furthermore, only eight genes were included in the study and therefore additional genes that contribute more strongly to modulating lipid levels may have been overlooked. However, a GWAS for lipid levels is underway for the entire AWI-Gen dataset. The Bonferroni method to reduce false discovery rate assumes that pairwise tests are independent and this is considered by many to be overly conservative, as many SNVs from the same gene locus would be in linkage disequilibrium. Therefore results from sets of tests may not be independent (Lewis, 2002). Lipid lowering medications may affect the direction of associations and mask the genetic effect on lipid levels, but since no person reported taking such medication this was not a concern for the study. Another limitation is that the study used SNV arrays, whereas sequencing data could have led to the discovery of new variants associated with lipid levels among Africans. We are mindful of the potential masking effect of comorbidity in our study sample on the signals of association with lipid levels. Further research is needed on gene-gene interaction and gene-environment interaction to fully elucidate the factors influencing lipid levels in this African population.

## Conclusion

Our findings showed that several variants in *CETP*, *LCAT*, *PCSK9*, and *PON1* were significantly associated with HDL-C, LDL-C and TC with the strongest signal being that of rs117231520 (*CETP*) with serum HDL-C levels in this Ghanaian population. *CETP* and *PCSK9* variants replicate previously reported lipid trait GWAS loci in African Americans and strengthen the evidence of the influence of these signals on lipid levels in peoples of African descent. The novelty of this study lies in the identification of three genetic polymorphisms in LD (rs854558, rs854564, and rs854565) in *PON1* which were associated with increasing TC levels in Ghanaians.

## Data Availability Statement

The data is available under the AWIGen Study in EGA (EGAS00001002482).

## Ethics Statement

This study was carried out in accordance with the recommendations of the National Institutes of Health guidelines, Human Research Ethics Committees (HREC) of the University of the Witwatersrand (ID No. M12109), the Ghana Health Service Ethics Review Committee (ID No. GHS-ERC:05/05/2015) and the Navrongo Health Research Centre Institutional Review Board (ID No. NHRCIRB178) with community engagement and written informed consent from all subjects. All subjects gave written informed consent in accordance with the Declaration of Helsinki. The protocol was approved by the Human Research Ethics Committee (HREC) of the University of the Witwatersrand (ID No. M12109), the Ghana Health Service Ethics Review Committee (ID No. GHS-ERC:05/05/2015) and the Navrongo Health Research Centre Institutional Review Board (ID No. NHRCIRB178).

## Author Contributions

GA, LA-E, EN, CD, and AO collected the data. AC performed genetic data curation. GA conducted the data analysis and drafted the manuscript. GA, LA-E, NC, and MR interpreted the results. AB and CR performed the replication assessment. LA-E, EN, CD, AO, NC, AC, AB, CR, and MR edited the draft. MR was the principal investigator and team leader in the AWI-Gen project and provided scientific leadership in the development of the research protocol. All authors read and approved the final draft.

## Conflict of Interest

The authors declare that the research was conducted in the absence of any commercial or financial relationships that could be construed as a potential conflict of interest. The handling Editor SO-A declared a shared affiliation, though no other collaboration, with one of the authors LA-E.

## Footnotes

1 http://pngu.mgh.harvard.edu/purcell/plink/

2 https://www.ncbi.nlm.nih.gov/dbvar

3 http://genome.sph.umich.edu/wiki/EPACTS

## Supplementary Material

Supplementary Figure 1 Principal component analysis using data from the H3Africa SNV array (∼2.3 million SNVs) on the full AWI-Gen dataset, including the participants from each of the six study sites. This study was based only on data from the Navrongo study site (nvr) and the participants clustered closely together illustrating that there was little population structure in this group. The other five study sites were Nanoro in Burkina Faso (nan), Nairobi (nai) and three study sites in South Africa: Soweto (SWT), Agincourt (agt) and Dikgale (dkg).
