# Supplementary figures and images for: Candidate Gene Analysis Reveals Strong Association of CETP Variants With High Density Lipoprotein Cholesterol and PCSK9 Variants With Low Density Lipoprotein Cholesterol in Ghanaian Adults: An AWI-Gen Sub-Study
Source: Front Genet. 2020 Oct 30;11:456661. doi: 10.3389/fgene.2020.456661 (PMC7661969; doi:10.3389/fgene.2020.456661)

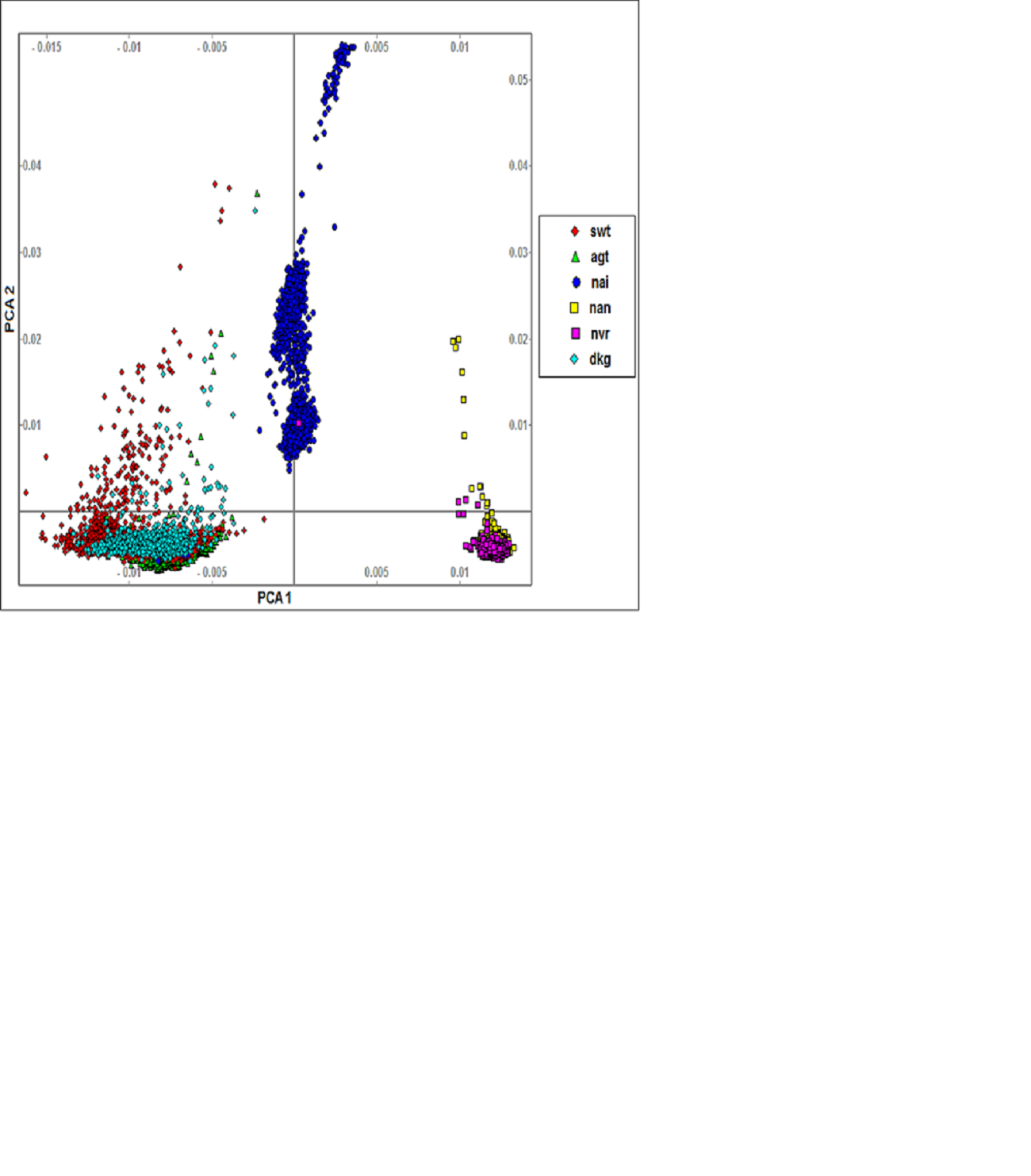

Supplement: Supplementary Figure 1 — Principal component analysis using data from the H3Africa SNV array (∼2.3 million SNVs) on the full AWI-Gen dataset, including the participants from each of the six study sites. This study was based only on data from the Navrongo study site (nvr) and the participants clustered closely together illustrating that there was little population structure in this group. The other five study sites were Nanoro in Burkina Faso (nan), Nairobi (nai) and three study sites in South Africa: Soweto (SWT), Agincourt (agt) and Dikgale (dkg). [file Image_1.TIF]
