# Supplementary material for: Candidate Gene Analysis Reveals Strong Association of CETP Variants With High Density Lipoprotein Cholesterol and PCSK9 Variants With Low Density Lipoprotein Cholesterol in Ghanaian Adults: An AWI-Gen Sub-Study
Source: Front Genet. 2020 Oct 30;11:456661. doi: 10.3389/fgene.2020.456661 (PMC7661969; doi:10.3389/fgene.2020.456661)
Supplement: Supplementary file 2 [file Data_Sheet_1.docx]

**Table S1: R^2^ values of significant variants in *CETP* associated with HDL-C levels in Ghanaians**

| Rs number | rs17231520 | rs34065661 | rs3816117 | rs711752 | rs708272 | rs4784740 | rs891141 | rs891142 | rs891143 | rs158478 | rs289719 |
| --- | --- | --- | --- | --- | --- | --- | --- | --- | --- | --- | --- |
| rs17231520 | 1.000 | 0.990 | 0.058 | 0.268 | 0.268 | 0.468 | 0.273 | 0.420 | 0.446 | 0.007 | 0.013 |
| rs34065661 | 0.990 | 1.000 | 0.059 | 0.271 | 0.271 | 0.479 | 0.269 | 0.415 | 0.441 | 0.008 | 0.013 |
| rs3816117 | 0.058 | 0.059 | 1.000 | 0.222 | 0.222 | 0.005 | 0.011 | 0.001 | 0.002 | 0.007 | 0.079 |
| rs711752 | 0.268 | 0.271 | 0.222 | 1.000 | 1.000 | 0.102 | 0.161 | 0.085 | 0.088 | 0.011 | 0.000 |
| rs708272 | 0.268 | 0.271 | 0.222 | 1.000 | 1.000 | 0.102 | 0.161 | 0.085 | 0.088 | 0.011 | 0.000 |
| rs4784740 | 0.484 | 0.479 | 0.005 | 0.102 | 0.102 | 1.000 | 0.589 | 0.876 | 0.927 | 0.031 | 0.031 |
| rs891141 | 0.273 | 0.269 | 0.011 | 0.161 | 0.161 | 0.589 | 1.000 | 0.672 | 0.636 | 0.001 | 0.048 |
| rs891142 | 0.420 | 0.415 | 0.001 | 0.085 | 0.085 | 0.876 | 0.672 | 1.000 | 0.945 | 0.021 | 0.023 |
| rs891143 | 0.446 | 0.441 | 0.002 | 0.088 | 0.088 | 0.972 | 0.636 | 0.945 | 1.000 | 0.019 | 0.023 |
| rs158478 | 0.007 | 0.008 | 0.007 | 0.011 | 0.011 | 0.031 | 0.001 | 0.021 | 0.019 | 1.000 | 0.033 |
| rs289719 | 0.013 | 0.013 | 0.079 | 0.000 | 0.000 | 0.031 | 0.048 | 0.023 | 0.023 | 0.033 | 1.000 |

**Table S2: R^2^ values of significant variants in *PON1* associated with HDL-C levels in Ghanaians**

| Rs number | rs854558 | rs854564 | rs854565 |
| --- | --- | --- | --- |
| rs854558 | 1.000 | 0.993 | 0.993 |
| rs854564 | 0.993 | 1.000 | 1.000 |
| rs854565 | 0.993 | 1.000 | 1.000 |
